# Bioethanol Production from Brewers Spent Grains Using a Fungal Consolidated Bioprocessing (CBP) Approach

**DOI:** 10.1007/s12155-016-9782-7

**Published:** 2016-08-08

**Authors:** Stuart Wilkinson, Katherine A. Smart, Sue James, David J. Cook

**Affiliations:** 1 Brewing Science Section, Division of Food Sciences, The University of Nottingham, Sutton Bonington Campus, Loughborough, Leicestershire LE12 5RD UK; 2 SABMiller Plc, SABMiller House, Church Street West, Woking, Surrey GU21 6HS UK

**Keywords:** Brewers spent grains, Lignocellulosic ethanol, Consolidated bioprocessing, Simultaneous saccharification and fermentation, Brewery co-products, Fungi

## Abstract

Production of bioethanol from brewers spent grains (BSG) using consolidated bioprocessing (CBP) is reported. Each CBP system consists of a primary filamentous fungal species, which secretes the enzymes required to deconstruct biomass, paired with a secondary yeast species to ferment liberated sugars to ethanol. Interestingly, although several pairings of fungi were investigated, the sake fermentation system (*A. oryzae* and *S. cerevisiae* NCYC479) was found to yield the highest concentrations of ethanol (37 g/L of ethanol within 10 days). On this basis, 1 t of BSG (dry weight) would yield 94 kg of ethanol using 36 hL of water in the process. QRT-PCR analysis of selected carbohydrate degrading (CAZy) genes expressed by *A. oryzae* in the BSG sake system showed that hemicellulose was deconstructed first, followed by cellulose. One drawback of the CBP approach is lower ethanol productivity rates; however, it requires low energy and water inputs, and hence is worthy of further investigation and optimisation.

**Electronic supplementary material:**

The online version of this article (doi:10.1007/s12155-016-9782-7) contains supplementary material, which is available to authorized users.

## Introduction

The production of advanced biofuels (second generation biofuels) from lignocellulosic biomass has a number of technical difficulties associated with it due to the recalcitrant nature of the material [1]. A thermo-chemical pre-treatment of some form is usually required to enhance the subsequent enzymatic hydrolysis (or saccharification) yield [2]. This pre-treatment stage is considered to be the most energy intensive and expensive stage of conventional biofuel production [2]. The subsequent enzymatic saccharification stage incurs further costs in terms of commercial enzyme preparations containing cellulases and xylanases. Overall, lignocellulosic biofuel production is hindered by economic factors which currently limit its widespread, large-scale production [3]. As a consequence of these technical difficulties and the economic implications of this approach, researchers have investigated entirely biological alternatives, such as simultaneous saccharification and fermentation (SSF) and consolidated bioprocessing (CBP; [4]). CBP involves the conversion of lignocellulose into the required products in one step, without the addition of enzymes. Most attempts at CBP have utilised individual organisms, such as thermo-tolerant yeast strains [5] or bacteria (e.g. species of *Clostridia;* [6]). Frequently, a genetically modified (GM) approach has been used for expression or over-expression of lignocellulolytic enzymes. However, the usage of obligate anaerobic species such as *Clostridia* has numerous technical difficulties associated with it, not least due to its pathogenicity to humans. Also, the usage of GM organisms has additional implications and restrictions in many parts of the world.

The production of ethanol by filamentous fungal species, already capable of secreting lignocellulolytic enzymes, has been reported [4]. In the absence of any thermo-chemical pre-treatment, the purely biological deconstruction and saccharification method would likely require a wide variety of carbohydrate degrading (CAZy) and associated enzymes [7]. Considering the natural propensity of many fungal species to deconstruct lignocellulosic material in the wild, the present research evaluated the potential to produce ethanol via CBP using fungal consortia. In this approach, a primary organism, such as a filamentous fungus, is used to deconstruct the lignocellulosic material through secretion of its native lignocellulolytic enzymes. Subsequently, a secondary fungal species, such as an industrial yeast strain, can ferment any liberated monomeric sugars into ethanol. Various candidates for the primary fungus are known, which secrete the required arsenal of lignocellulolytic enzymes, including *Aspergillus niger*, *Aspergillus oryzae*, *Trichoderma reesei* and *Humicola insolens* [8]. The production of the Japanese alcoholic beverage sake utilises a consortium of *A. oryzae* and *S. cerevisiae* (NCYC479) to produce high concentrations of ethanol (ca. 20 % ABV) from the starch component found within rice [9]. *A. oryzae* is responsible for the secretion of the enzymes (primarily α-amylases and endo-1,4-α-d-glucan glucohydrolase EC 3.2.1.1) that hydrolyse the starch into glucose, which *S. cerevisiae* then utilises for ethanol production [10]. However *A. oryzae* has also been shown to secrete lignocellulolytic enzymes (endocellulases and various xylanases) in significant quantities when cultured in media containing lignocellulosic biomass [11]. The sake fermentation system was thus of interest to us as a potential CBP approach for lignocellulosic bioethanol production, particularly since *A. oryzae* and *S. cerevisiae* are known to exhibit suitably high ethanol tolerance phenotypes [12].

Advanced biofuel production using agricultural or industrial co-product processing streams containing lignocellulosic material is of particular interest because it avoids the human ‘food versus fuel’ dilemma of first-generation biofuel substrates [4]. Brewers spent grain (BSG) is a co-product of the brewing process which is abundant with ca. 9.9 million tons wet weight annually from the EU alone (calculation based upon beer production in the EU for 2014 at approximately 522.8 million hL [13] with the following assumptions: approximate global average malt requirement of 11.8 kg/hL, 22 % dry weight of malt inwards generating 1.35 million metric tonnes [dry weight] with ca. 78 % moisture content producing 6.1 million tonnes fresh weight and 19 kg [fresh weight] per hL). BSG is also typically sold at a very low cost of ca. £38 per tonne wet weight [14]. Within BSG, the hemicellulose and cellulose contents typically range from 10 to 25 % and 15 to 30 %, respectively (depending on the barley cultivar and brewery technology employed), providing a significant pool of potentially fermentable sugars. In addition, BSG typically contains between 15 and 27 % protein. In contrast to lignocellulosic biofuel substrates such as wheat straw or switchgrass, this renders BSG an ideal substrate for microbial growth due to the significant nitrogenous component (which facilitates the production and secretion of various enzymes). BSG has been used as a growth substrate for the cultivation of the fungus, *T. reesei*, for the production of various cellulase enzymes (endo and exoglucanases; [15]). In addition, BSG has also been used as a substrate for the cultivation of *A. oryzae* for the production of α-amylases. *A. oryzae* has also been shown to secrete various proteases when grown on wheat straw [16] indicating its potential to utilise the significant nitrogenous component found within BSG.

Here, we evaluate a CBP approach to ethanol production from BSG using consortia of various filamentous fungal species, each paired with a selected yeast strain. The sake fermentation system was included in the study for the reasons outlined above and is compared with alternative consortia in terms of ethanol productivity. In addition, the gene expression of a selection of key carbohydrate degrading (CAZy) enzymes and associated enzymes by *A. oryzae* was studied in the ‘sake fermentation’ of BSG, to try and identify which substrates were being utilised and at which stages of the fermentation.

## Materials and Methods

### Reagents

All reagents were of AR grade and obtained from Sigma-Aldrich (UK) and Fisher Scientific (UK). All water used was deionised by reverse osmosis and of ≥18 MΩ purity (Purite Select Ondeo IS water system Purite, UK).

### BSG

Brewers spent grains (BSG) were supplied by the SABMiller research brewery at the Sutton Bonington campus of the University of Nottingham. The BSG for this research was derived from high gravity brewing using 100 % barley malt. For the experiments using ground BSG, the material was oven dried overnight at 105 °C and ground to a particle size of less than 212 μm to ensure homogeneity (KG49 grinder, Delonghi, UK). For the lauter tun experiments, the BSG was directly removed from the lauter tun after completion of mashing and used with minimal delay. The composition of the BSG used in this study is given in Table 1.

**Table 1:** Composition of BSG used in the present study

BSG component	% (*w*/*w*)
Starch	1.2 ± 0.11
Protein	27.9 ± 0.18
Ash	2.7 ± 0.21
Lipid	6.3 ± 1.4
Lignin	10.7 ± 2.2
Cellulose (glucose)	22.1 ± 0.8
Hemicellulose	19.3 ± 1.8
Of which xylose	11.3 ± 1.2
Other	8.6

Data are the mean ± standard deviation of three replicate measurements

### Compositional Analysis of BSG

#### Analysis of the Total Glucose and Xylose Content of BSG

The total glucose (analogous to cellulose content) and xylose content of the BSG were quantified using the assay described by Wilkinson et al. [17]. Samples underwent complete acid hydrolysis (using 12 M H_2_SO_4_ at 37 °C for 1 h then diluted to 1 M for 2 h incubation at 100 °C and then subsequent quantification of liberated sugars by ion chromatography). Cellulose was calculated after stoichiometric correction for dehydration using the 0.9 multiplication factor [18].

#### Hemicellulose Quantification

Hemicellulose was quantified using a gravimetric method outlined in Wilkinson et al. [19] after digestion with 4 M KOH for 2 h. The samples were then filtered and 80 % acetone solution added followed by centrifugation at 3500 rpm for 5 min. The supernatant was discarded and the pellet exhaustively washed in 95 % ethanol before centrifugation at 3500 rpm for 5 min. The final pellet was then dried overnight at 45 °C prior to weighing.

#### Total Solvent Extractable Lipid Analysis

Lipid analysis was conducted according to the method outlined in Wilkinson et al. [19]. A dichloromethane/methanol mixture (2:1, *v*/*v*) was added to samples followed by agitation for 2 h using a roller bed. The aqueous upper phase was then removed and the lower organic phase (contained the extracted lipid) was then dried overnight at 40 °C prior to gravimetric determination.

#### Lignin Quantification

Acetyl bromide soluble lignin (ABSL) was quantified using the method outlined in Wilkinson et al. [20]. Samples were incubated with 25 % acetyl bromide solution (prepared in glacial acetic acid) at 50 °C for 2 h. Quantification was then performed by spectrophotometric measurements (taken at 280 nm) and comparison to authentic lignin standards.

#### Measurement of the Protein Content

A Thermo Flash Nitrogen Analyser (ThermoFisher Scientific, Waltham, Massachusetts, USA) was used to determine protein content according to the method outlined in Wilkinson et al. [19]. An initial combustion temperature of 900 °C was used which was then raised to 1800 °C, and the reduction reactor was kept at 680 °C. Protein was determined using the N × 6.25 conversion factor.

#### Starch Quantification

Starch was quantified using a glucose oxidase/peroxidase kit (GAGO-20, Sigma-Aldrich) according to the manufacturer’s instructions.

#### Ash Analysis

This was conducted using the method outlined in Wilkinson et al. [19]. Samples were placed into a muffle furnace at 580 °C for 24 h until a constant weight was achieved.

### CBP and Culture of Organisms

All CBP experiments were conducted using 50 g of dried and ground BSG (unless otherwise stated) suspended in 200 mL of water, inoculated with both the primary filamentous fungal and secondary yeast species (both on day 0) and then incubated at the required temperature for approximately 20 days. Samples of the supernatant were then taken for analysis (monosaccharide liberation, ethanol production and assessment of lignocellulolytic enzyme activity) at various intervals. The efficacy of each CBP system was then evaluated in terms of maximal ethanol concentration generated and volumetric productivity (g/L/day). All experiments were conducted in triplicate.

*Saccharomyces cerevisiae* strains NCYC479 and NCYC2592 were chosen as partners for the primary fungal species due to their high ethanol tolerance phenotypes. The standard sake system used the NCYC479 sake yeast strain. *Kluyveromyces* strains were also evaluated as a secondary strain due to their potential for pentose sugar utilisation (both for cellular metabolism and possibly for fermentation to ethanol). Here, we evaluated the use of *K. marxianus* strains NCYC1426 and NCYC179 and *K. wickerhamii* strain NCYC546. All strains were obtained from NCYC (National Collection of Yeast Cultures, UK). Sequential 3-stage propagation (using 4 % YPD media and the method described in Wilkinson et al.) [20] was employed for each yeast strain in order to culture a sufficient cell density for the required number of fermentation vessels. All CBP experiments were inoculated with the required yeast strain at a concentration of approximately 10^6^ (viable) cells/mL.

#### CBP Experiments

##### CBP Experiments Utilising *A. oryzae* as the Primary Filamentous Fungal Species

The efficacy of the sake system was evaluated on BSG in a variety of forms in order to better understand the effects of substrate form and pre-treatment. A comparison was made between (i) 50 g dried and ground BSG (the ‘standard’ protocol), (ii) 50 g BSG which had been subjected to 1 % *v*/*v* HCl 121 °C hydrothermal pre-treatment and (iii) 50 g ‘wet’ BSG direct from the lauter tun (dry weight, corrected for moisture content which was determined by drying to constant weight at 105 °C (HR83.P moisture analyser, Mettler Toledo, UK). Pre-treatment of BSG was conducted using the optimised protocol described in Wilkinson et al. [19], namely 1 % HCl at 121 °C for 30 min (at 25 % *w*/*v* solids loading) using a 40-L benchtop autoclave (Priorclave, Tactrol 2, RSC/E, UK). This pre-treatment was previously shown to effectively enhance enzymatic saccharification yields (when subsequently using cellulolytic enzymes) without excessively degrading the sugars, as minimal concentrations of furan-based inhibitors were generated. This was further confirmed when the feedstock produced from this pre-treatment was ultimately used to efficiently produce ethanol using the NCYC479 strain of *S. cerevisiae*.

Supplementary experiments were conducted with variants of the sake system using ground BSG only. These included the use of *S. cerevisiae* NCYC 2592 in place of the sake strain (NCYC 479) and also separate experiments whereby 1 mL Cellic® CTec2 (kindly supplied by Novozymes A/S, Demark) was added into each vessel on day 10, to establish whether cellulolytic enzyme secretion from the primary fungal species was a limiting factor in the production of ethanol. The CTec2 was dosed at 10 FPU/g biomass, which was determined according to Ghose [21]. Day 10 was chosen for the addition of the enzymes, as preliminary work concluded that maximal ethanol yields were achieved by this point.

An additional ‘hybrid’ sake system was also investigated in parallel experiments but with the addition initially of 1.0 ± 0.1 g of pre-cultured *H. insolens* filamentous fungal biomass (propagation described in Sect. 2.5.4) and then incubated for 20 days at either 15 or 30 °C.

The pairing of *Kluyveromyces* spp. with *A. oryzae* was also evaluated. Experiments were conducted as previously (ground BSG substrate) but using either *K. marxianus* NCYC1426, *K*. *marxianus* NCYC179 or *K*. *wickerhamii* NCYC546 as the secondary yeast strain. In addition, consortia featuring each individual *Kluyveromyces* strain together with *S. cerevisiae* NCYC479 were also tested. These fermentations were inoculated with just *S. cerevisiae* NCYC479 on day 0 and incubated at 15 °C until day 10 (targeting the fermentation of hexose sugars). On day 10, the vessels were then inoculated with the desired *Kluyveromyces* spp. Additional aeration was also provided from day 10 onwards in order to encourage the aerobic-based pentose metabolism of the *Kluyveromyces* spp. Enzyme-supplemented variants of these systems were also conducted using Cellic® CTec2 dosed on day 10 as previously described. Lastly, sake CBP cultures were prepared as previously described (i.e. with the koji mould; *A. oryzae* and also *S. cerevisiae*) except without the addition of any BSG (koji controls). Any ethanol subsequently produced in these controls was then subtracted from the ethanol yields achieved in experiments on BSG in order to compensate for the fermentation of any starch-based glucose which was present in the koji.

#### CBP Utilising *A. niger* with either *S. cerevisiae* NCYC2592 or NCYC479

Spores of *A. niger* (N402) were incubated on PDA slopes (potato dextrose agar; Oxoid) supplemented with 10 mM uridine (Sigma-Aldrich) in vials at 28 °C with agitation at 120 rpm (MaxQ 4358 shaking incubator, Thermo Scientific, UK) until they had conidiated. The spores were harvested prior to inoculation by harvesting into 0.1 % (*v*/*v*) Tween 20. All the experimental vessels contained 50 g ground BSG and 200 mL water and were inoculated with *A. niger* spores to a concentration of approximately 10^7^ spores/mL (considered to be an excess of spores) and then inoculated with either *S. cerevisiae* NCYC2592 or NCYC479 at a concentration of approximately 10^6^ viable cells/mL. The vessels were then incubated at 25 °C for 17 days.

#### CBP Using *Humicola insolens* with either *S. cerevisiae* NCYC2592 or NCYC479

Spores of Humicola were incubated in YpSs media containing 1.5 % Bacto soluble starch, 0.4 % yeast extract, 0.1 % dipotassium phosphate, and 0.05 % magnesium sulphate (Oxoid, UK) in vials at 40 °C in the dark until they had germinated. All the experimental vessels contained 50 g dried, ground BSG and 200 mL water and were inoculated with 1.0 g ± 0.1 of *H. insolens* filamentous tissue and then inoculated with either *S. cerevisiae* NCYC2592 or NCYC479 at a concentration of ca. 10^6^ viable cells/mL. Cultures were incubated at 30 °C for 20 days. This temperature was chosen as a compromise between the 40–45 °C optimum growth condition for *H. insolens* and the need to ensure viability of the *S. cerevisiae* strains.

#### Semi-Quantitative Assay for Evaluation of the Secreted Cellulase and Xylanase Activities in Supernatants of the CBP Systems

The detection of any cellulase or xylanase activity in the supernatants of each of the CBP experiments was achieved using the Congo red staining method [22, 23].

### QRT-PCR Analysis of CAZy Gene Expression in *A. oryzae* when Used in the Sake CBP System

A standard sake CBP protocol was conducted (as previously described in Sect. 2.4.1.1) with sacrificial replicates for sampling on days 1, 5, 10 and 15 (each time point performed in triplicate). Separation of the liquid phase (supernatant) from the solid phase was conducted via filtration using Miracloth (Merck KGaA, Darmstadt, Germany). The solid and liquid samples were each separately flash-frozen in liquid nitrogen and then stored at −80 °C until required.

All PCR primers were designed using the Eurofins Primer Design Tool and synthesised by Eurofins (MWG Operon, Germany) from target candidate genes identified from the genome databases of *Aspergillus oryzae* (http://www.aspgd.org/) and *Saccharomyces* (http://www.yeastgenome.org/). The ACT gene which encoded actin [24] was used as the housekeeping gene for expression levels to compare with the target CAZy genes. After successful confirmation that the designed primers were specific to *A. oryzae* (and not *S. cerevisiae*), total RNA was extracted from the frozen samples (from the 20-day fermentations using the sake-based CBP system) and purified using a fungal RNA purification kit for *A. oryzae* (Norgen Biotek Plant/Fungi Total RNA Purification Kit, Canada) according to the manufacturer’s instructions. All extracted RNA concentrations were then quantified using a NanoVue™ microvolume spectrophotometer at both 230 and 260 nm (GE healthcare, USA). First-strand complementary DNA (cDNA) synthesis from the extracted RNA was then performed using a first-strand cDNA synthesis kit (GE healthcare, USA) according to the manufacturer’s instructions. Quantitative RT-PCR was then performed with a StepOnePlus real-time PCR system (Applied Biosystems, USA) using Fast SYBR®green mastermix (Applied Biosystems, USA) according to the manufacturer’s guidelines. Running conditions were as follows: 40 cycles of 94 °C for 1 min (denaturation), 60 °C for 30 s (primer annealing) and 72 °C for 30 s (polymerisation).

### HPLC

Ethanol concentrations were quantified using high-performance liquid chromatography (HPLC) using a Rezex ROA column (Phenomenex, UK) with refractive index (RI) detection using the method described in Wilkinson et al. [19]. Liberated sugars (in the supernatant) were quantified via ion-chromatography (IC) using a Carbo-Pac PA20 column (Dionex, USA) with pulsed amperometric electrochemical detection (PAD) using the method described in Wilkinson et al. [19].

### Measurement of the Protein Content of Solid Residues Remaining after CBP Fermentation of BSG

A Thermo Flash Nitrogen Analyser (ThermoFisher Scientific, Waltham, Massachusetts, USA) was used to determine protein content using the method described in Wilkinson et al. [19]. All analyses were conducted in triplicate.

## Results and Discussion

### CBP Using *A. oryzae* with *S. cerevisiae* NCYC479 (Sake-Based CBP System)

**Fig. 1:**
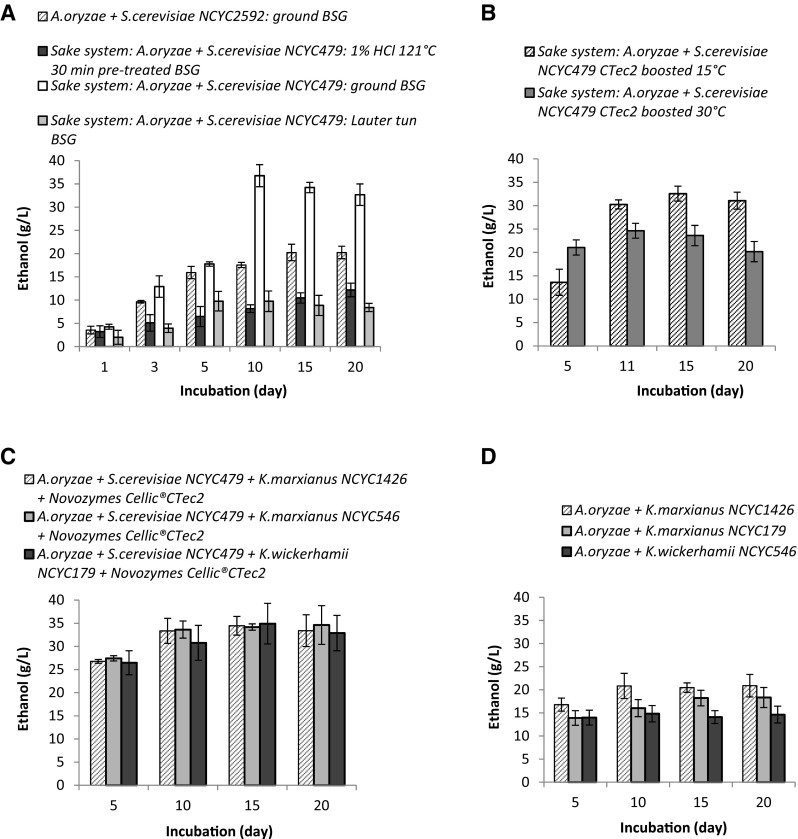
Time course of ethanol production from BSG using consolidated bioprocessing with fungal consortia under the specified conditions (see data series legends). **a** At 15 °C. **b** At both 15 and 30 °C using the sake system but with the addition of Novozymes Cellic® CTec2 on day 10 (10 FPU/g biomass). **c** At 15 °C and inoculated initially with *S. cerevisiae* NCYC479 on day 0 and then subsequently inoculated with *Kluyveromyces* spp. and boosted with Novozymes Cellic® CTec2 on day 10 (dosed at 10 FPU/g biomass). **d** *A. oryzae* in partnership with three different strains of *Kluyveromyces*: *marxianus* NCYC1426, *marxianus* NCYC179 and *wickerhamii* NCYC546 at 15 °C

Of all the pairings of primary filamentous fungi and secondary yeast strains which were tested, the sake pairing (*A. oryzae* and *S. cerevisiae NCYC479*) was by far the most effective in terms of ethanol production (Table 2). Relatively high ethanol concentrations of up to 37 g/L (ca. 4 % ABV) were attained using this system with 10-days incubation at 15 °C, on a substrate of dried and ground BSG (Fig. 1a). The data in Table 2 are ordered by increasing ethanol yield; hence, it can be readily noted that the eight best ethanol-yielding systems all contained both *A. oryzae* and *S. cerevisiae NCYC479*. When BSG direct from a lauter tun was used as substrate, without prior drying and milling, ethanol yields were significantly lower (at best ca. 9.8 g/L after 5 days). This highlighted the requirement for some form of particle size reduction of the BSG in order to enhance the saccharification of the lignocellulosic material by the enzymes secreted from the *A. oryzae*. A reduction in particle size may also allow greater penetration of the substrate by fungal hyphae, as described in Lee [25], thus facilitating more successful interaction between the secreted enzymes and the substrate. However, conversely, the relatively high temperature drying process (105 °C) that occurred prior to the particle size reduction could have actually had a negative effect on sugar liberation from cellulose (and therefore also affected the ethanol yields). This is through the collapse of the cellulose pores which may have ultimately impeded enzymatic access somewhat. The superior ethanol yields from the sake-based CBP system (relative to other permutations of fungal species tested) were somewhat unexpected, since *A. oryzae* is not usually considered a cellulolytic fungal species, being more noted for starch hydrolysis [10]. Semi-quantitative analysis of secreted enzyme activities, using the congo red staining method, indicated significant cellulase and xylanase activity in the supernatants generated from 5-days incubation and onwards.

**Table 2:** Maximal ethanol yields (g/L) and volumetric productivity (g/L/day) achieved from all CBP variants tested. Data are the mean of three replicate experiments

CBP/SSF system	Mean maximal ethanol yield (g/L)	Volumetric productivity^a^ (g/L/day)
*A. niger + S. cerevisiae* NCYC2592	0.7 ± 0.1	0.1
*A. niger + S. cerevisiae* NCYC479	6.7 ± 0.4	2.3
*H. insolens + S. cerevisiae* NCYC2592	8.7 ± 0.5	0.9
*A. oryzae + S. cerevisiae* NCYC479 (BSG direct from lauter tun)	9.8 ± 2.2	1.0
*A. oryzae + S. cerevisiae* NCYC479 (1 % HCl 121 °C pre-treated BSG)	12.2 ± 0.8	1.2
*A. oryzae + Kluyveromyces* spp. NCYC546	14.8 ± 1.4	1.5
*A. oryzae + Kluyveromyces* spp. NCYC546 + Novozymes Cellic®CTec2	16.5 ± 3.4	1.7
*A. oryzae + Kluyveromyces* spp. NCYC179	18.2 ± 1.7	1.8
*A. oryzae + Kluyveromyces* spp. NCYC179 + Novozymes Cellic®CTec2	18.3 ± 3.7	1.8
*H. insolens + S. cerevisiae* NCYC479	18.5 ± 0.1	1.9
*A. oryzae + S. cerevisiae* NCYC2592	20.3 ± 0.6	2.0
*A. oryzae + Kluyveromyces* spp. NCYC1426	20.8 ± 1.0	2.1
*A. oryzae + Kluyveromyces* spp. NCYC1426 + Novozymes Cellic®CTec2	22.6 ± 2.0	2.3
*A. oryzae + S. cerevisiae* NCYC479 + Novozymes Cellic®CTec2 (30 °C)	24.6 ± 1.6	2.5
*A. oryzae + S. cerevisiae* NCYC479 + *Kluyveromyces* spp. NCYC1426 + Novozymes Cellic®CTec2	25.0 ± 2.0	2.5
*A. oryzae + S. cerevisiae* NCYC479 + *Kluyveromyces* spp. NCYC179 + Novozymes Cellic®CTec2	26.1 ± 4.0	2.6
*A. oryzae + S. cerevisiae* NCYC479 + *Kluyveromyces* spp. NCYC546 + Novozymes Cellic®CTec2	26.2 ± 4.4	2.6
*A. oryzae + H. insolens + S. cerevisiae* NCYC479 (30 °C)	30.0 ± 0.1	3.0
*A. oryzae + S. cerevisiae* NCYC479 + Novozymes Cellic®CTec2 (15 °C)	32.6 ± 1.6	3.3
*A. oryzae + H. insolens + S. cerevisiae* NCYC479 (15 °C)	33.0 ± 1.4	3.3
*A. oryzae + S. cerevisiae* NCYC479	36.8 ± 2.4	3.7

All BSG was dried and ground unless otherwise stated

^a^Volumetric productivity calculations based on number of days taken to achieve highest mean ethanol yields for all CBP systems tested

No sugars (monosaccharides or oligosaccharides less than Dp5) were detected in any supernatant samples (when using either secondary yeast strain partner) indicating that any sugar liberated from the lignocellulosic material was likely occurring in very small trace amounts and was being utilised immediately by either the yeast species or the filamentous fungus. The rapid utilisation of any liberated sugars by the yeast species (the *S. cerevisiae*) could possibly limit catabolite repression or product inhibition of any enzymes secreted by the primary deconstructive organism (the fungus *A. oryzae* in this case) and may also further induce enzyme secretion from the fungus due to the lack of immediately available carbon sources. This is similar to the phenomenon described in Suto and Tomita [26]. Although the IC analysis could not detect oligosaccharides greater than Dp5, it was also considered unlikely that any of the fungi used in these CBP systems would have been able to directly utilise these larger molecular weight sugars in the supernatant. Further hydrolysis of these cellodextrins to lower molecular weight sugars such as glucose would be a more probable mechanism of carbon source utilisation for both the filamentous fungi and yeast. A very small quantity of ethanol was produced in the koji controls (i.e. vessels containing no BSG; with just the koji *A. oryzae* inoculum and *S. cerevisiae*) which could be attributed to hydrolysis of the very small quantity of starch that was present in the koji inoculum. However, this amount was subtracted from the final ethanol yields generated so as to allow for accurate evaluation of the degree of usage of the BSG carbon source. Therefore, the presence of a small quantity of starch contributed by both the koji and also in the BSG could have facilitated the initial growth of *A. oryzae* (as a rapidly utilisable carbon source favoured by the *A. oryzae* with its strong arsenal of amylose degrading enzymes) which then subsequently encouraged greater lignocellulosic enzyme secretion once the media was depleted of more readily available carbon sources. The small quantity of starch present in the BSG (ca. 1 % *w*/*w*) was also only considered to potentially contribute a small quantity of the carbon flux to ethanol. This was calculated to potentially offer a maximum contribution of approximately 1.5 g/L ethanol (of the total produced). Therefore, when considering the high ethanol yields achieved (ca. 37 g/L optimally), it is clear that starch (as the carbon source alone) could not explain the ethanol produced and a significant contribution was likely provided by the lignocellulosic material.

Ethanol yields from BSG using the sake-fermentation system were compared at 15 and 30 °C. Similar ethanol concentrations resulted in each case, with the higher temperature giving slightly higher ethanol concentrations at the 5-day time point (ca. 19 g/L ethanol compared to ca. 15 g/L at 15 °C: Supplementary Fig. 1). However, over longer timescales, from day 11 onwards, the 15 °C fermentations generated between 6 and 30 % more ethanol than fermentations at 30 °C. There could perhaps have been a slight increase in the rate and degree of evaporative loss of ethanol at 30 °C relative to 15 °C, which went some way to explaining this. Overall, these results indicated that the process has the potential to function effectively over a wide temperature range, without close regulation of temperature (potentially obviating the need for heating or cooling control). The process might therefore be carried out under ambient conditions in many countries.

Interestingly, thermochemical pre-treatment of BSG (1 % HCl, 121 °C, 30 min) reduced ethanol yields in the sake-fermentation system (Fig. 1a). Just 12 g/L ethanol was attained after 20-day incubation, representing around one third of the yield from non-pre-treated BSG. This system also took longer to achieve maximal ethanol concentrations (around 20 days, as opposed 10 days for the non-pre-treated BSG). Moderate cellulase activity was the only enzyme activity detected in the supernatants when using pre-treated BSG. The absence of any xylanase activity was not surprising, as the acid catalysed hydrothermal pre-treatment was likely to have solubilised the majority of hemicellulose present in the starting material, leaving cellulose as the only structural polysaccharide present in significant quantity, as suggested by Wilkinson et al. [19]. However, the degree of cellulase activity observed in the supernatants when using pre-treated BSG was lower than that observed with non-pre-treated BSG, which suggests that the original lignocellulosic material is a better activator of cellulase enzyme secretion from *A. oryzae*. Assumedly, the native, non-modified structure of the lignocellulose is more successfully recognised by *A. oryzae*, or results in more successful enzyme-substrate interactions, or both. In addition, the recognition of other lignocellulosic components such as hemicellulose might be crucial in triggering optimal secretion of cellulases through some form of signalling. This could be similar to the importance of the XLR-1 xylan degradation regulator (found in an alternative fungal species *Neurospora crassa*) with regard to its requirement of induction of other cellulolytic encoding genes [5]. Retaining the *A. oryzae* fungal species but substituting the original *S. cerevisiae* NCYC479 yeast strain partner with the NCYC2592 strain resulted in maximal ethanol concentrations of only ca. 20 g/L after 15 days (Fig. 1a). This equated to ca. 45 % lower maximal ethanol yields than when using the NCYC479 yeast. Whilst moderate cellulase and xylanase enzyme activities were detected in the supernatants at various time points, these were determined to be less than were observed with *S. cerevisiae* NCYC479, which might explain the lower observed ethanol yield.

The impact of adding cellulolytic enzymes (Cellic® CTec2, 10 FPU/g biomass) to the Sake fermentation system in an attempt to increase ethanol yield and productivity rate was investigated (Fig. 1b). However, no increase in ethanol yield was achieved with addition of supplementary enzymes (compare Fig. 1a, b), which suggests that cellulolytic enzyme secretion by *A. oryzae* was not a rate-limiting factor in terms of ethanol productivity by *S. cerevisiae*. It may be that the actual activity of endogenous secreted enzymes is mass transfer limited, for example due to the relatively high viscosity of the media. Operation of the sake-based CBP system at a lower solids loading (lower solid to liquid ratio) or the use of stirred or mixed fermentation vessels could possibly result in higher ethanol yields, by increasing successful enzyme-substrate interactions. Paradoxically, however, enzyme secretion has been shown to be greater on solid-state cultures as compared with highly dilute liquid cultures [27]. This could perhaps relate to a starvation response, as seen in biofilm formation. Once again, lower ethanol yields were achieved from fermentations at 30 °C compared to 15 °C (even with the additional cellulolytic enzyme supplementation; Fig. 1b). Cellic® CTec2 displays optimum activity in the region of 50 °C [28]; hence, the higher temperature fermentation might have been expected to perform better, from that perspective. That this was not the case once again suggests that the enzyme concentration was not the rate-limiting factor in these fermentations. It has previously been reported that temperature reduction can play a role in maintaining continued expression of extracellular hydrolases when using *A. oryzae* in a solid-state cultivation system for the traditional manufacture of products such as soy sauce [27]. The hypothesis, which may help to explain the results observed here, is that reduced mobility of the secreted enzymes in solid-state cultures at lower temperatures may significantly reduce product feedback inhibition of the expression of hydrolases, therefore resulting in higher secreted enzyme concentrations.

Analysis of xylose in the supernatants from both the 15 and 30 °C enzyme-supplemented sake systems indicated a significantly higher concentration (up to 5 g/L was present), as compared to the equivalent non-enzyme supplemented fermentations (up to 1 g/L; supplementary data Fig. 2). Hence, the xylanases present in the Novozymes Cellic® CTec2 hydrolysed an additional proportion of the hemicellulose present in the BSG; however, this increased hemicellulose hydrolysis did not significantly improve the final ethanol yields achieved.

### Evaluation of *Kluyveromyces* Yeast Strains in CBP of BSG for Ethanol Production

Whilst many different permutations were tested (Fig. 1c, d; Table 2), the use of *Kluyveromyces* spp., either alone with *A. oryzae* (i.e. without *S. cerevisiae*) or in a triple consortium with both the *A. oryzae* and *S. cerevisiae* NCYC479, resulted in all cases in lower ethanol yields than the standard sake-based system of *A. oryzae* with *S. cerevisiae* NCYC479 alone. All three *Kluyveromyces* species (*K*. *marxianus* NCYC1426, *K*. *marxianus* NCYC179 and *K*. *wickerhamii* NCYC546) performed similarly in terms of ethanol yields (Figs. 1c, d and Supplementary Fig. 3).

In an attempt to further optimise the fermentation of C-5 sugars by *Kluyveromyces* spp., experiments were run using the standard sake-CBP system to ferment C-6 sugars up until day 10, after which Cellic CTec2 (10 FPU/g biomass) and *Kluyveromyces* species were added under aerobic conditions; this approach produced less than 35 g/L ethanol (Fig. 1c). These yields were again inferior to the standard sake-based CBP system. Purely *Kluyveromyces*-based CBP combinations with *A. oryzae* produced ca. 15–20 g/L maximal ethanol concentrations (Fig. 1d). Supplementation with Cellic® CTec 2 (using only *Kluyveromyces* yeast strains) once again failed to increase ethanol yields relative to non-supplemented systems, further supporting the earlier observation that enzyme secretion by *A. oryzae* did not appear to be rate-limiting (Supplementary Fig. 3).

### CBP Using a Consortia of *H. insolens*, *A. oryzae* and *S. cerevisiae* NCYC479 (Hybrid Sake-Based System)

**Fig. 2:**
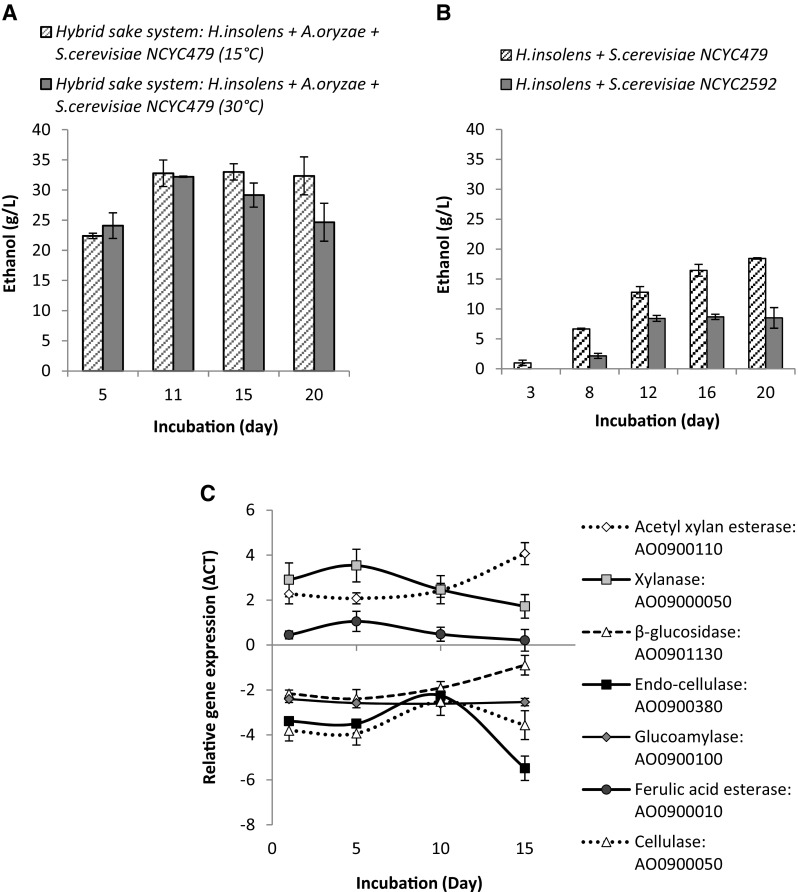
Time course of ethanol production from BSG using consolidated bioprocessing with fungal consortia under the specified conditions (see data series legends) **a** at both 15 and 30 °C using a consortium of *H. insolens*, *A. oryzae* and *S. cerevisiae* NCYC479. **b** At 30 °C using a consortium of *H. insolens* and two individual strains of *S. cerevisiae* (NCYC2592 or NCYC479) and **c** qRT-PCR analysis of gene expression levels versus time (relative to ACT housekeeping gene) for seven *A. oryzae* target genes (CAZy and associated genes used to indicate carbon source utilisation) in the sake CBP system grown on BSG

In these experiments, *H. insolens* was used in addition to the sake fermentation system, to see whether its enzyme secretion could boost ethanol production. However, ethanol yields were broadly similar at most time points (ca. 25–35 g/L ethanol at either 15 °C or 30 °C; Fig. 2a) to those for the standard sake system, indicating that the presence of the *H. insolens* did not significantly improve ethanol productivity. However, there was evidence of faster initial production of ethanol in this system. At the 5-day time-point, there was a small increase in ethanol concentration in the presence of *H. insolens* (16 and 36 % higher ethanol yields for the 15 and 30 °C fermentations, respectively) compared to the standard sake system. This might be due to the thermophilic nature of *H. insolens* (45 °C optimal) and production of additional deconstructive enzymes. For any commercial application of this fermentation system, an evaluation would need to be made to establish whether the day 5 ethanol yield improvement (specifically the volumetric productivity) was sufficient to justify inclusion of the *H. insolens*.

### CBP Using *A. niger* with either *S. cerevisiae* NCYC2592 or NCYC479

*A. niger* is in theory an excellent CBP primary fungal candidate since it possesses a large arsenal of lignocellulose degrading enzymes [29] and is used in commercial biotechnology to produce enzymes due to its high capacity secretory system [30]. It was therefore expected to perform well in terms of the production of relatively high concentrations of ethanol when partnered with a suitable *S. cerevisiae* strain (i.e. either NCYC2592 or NCYC479). However, only partnership with NCYC479 generated significant amounts of ethanol (maximum yield 6.7 g/L after just 6 days; supplementary Fig. 4), and both systems were much lower yielding than the sake system at equivalent time-points. Only a moderate degree of activity of both cellulases and xylanases was present in the supernatant from the NCYC479 experiments.

### CBP Using *H. insolens* with either *S. cerevisiae* NCYC2592 or NCYC479

Both secondary yeast strains (*S. cerevisiae* NCYC2592 & NCYC479) in partnership with *H. insolens* successfully produced ethanol directly from BSG and with strain NCYC479 again significantly out-performing NCYC2592 (maximal ethanol concentration of ca. 20 g/L by day 12 compared to only ca. 8.5 g/L at the equivalent stage with NCYC2592; Fig. 2b). Semi-quantitative analysis of enzyme activity indicated a greater activity of both cellulase and in particular xylanase activity in the supernatant produced using NCYC479, as compared to NCYC2592. In addition to the higher ethanol yields which were achieved with the NCYC479 yeast strain, a significant increase in filamentous fungal biomass production was noted (5.6 g ± 1.2 g of *H. insolens* fungal biomass (dry weight) compared to only 1.3 g ± 0.4 g biomass in the NCYC2592 system). This large increase in fungal biomass, apparently purely in response to the inclusion of a different yeast partner, is not readily explained. Perhaps, the NCYC479 strain did not deplete (and therefore rate limit) a particular micronutrient that was essential for fungal biomass generation.

### QRT-PCR Analysis of CAZy Gene Expression in *A. oryzae* when Using the Sake CBP System for Ethanol Production from BSG

Amongst the first genes monitored which were up-regulated was the xylanase (AO090005001210) which peaked in activity at around day 5, beyond which its expression began to decline (Fig. 2c). This suggests that the *A. oryzae* targeted the hemicellulose (xylan) for degradation first, possibly due to its lower recalcitrance compared to that of crystalline cellulose or lignin. This is similar to the pattern (expression sequence of CAZy genes) seen by Delmas et al. [31], who used next-generation RNA sequencing technology (RNA-seq) in a study with *A. niger* cultured on wheat straw. It seems that in the absence of a preferred carbon source (glucose or starch), both *A. oryza*e and *A. niger* preferentially degrade hemicelluloses, perhaps as a pre-requisite of breaking down the lignocellulosic structure. Some degree of up-regulation of the ferulic acid esterase gene (*faeB*: AO090001000066) was indicated at an early stage, peaking at ca. day 5. Either the liberated hydroxycinnamic acids (predominantly ferulic acid) were being used by *A. oryzae* as a carbon source, or alternatively, cleavage of the di- and tri-ferulic acid esterified cross linkages was a key step in breaking down the lignocellulosic matrix and thus improving the access or performance of other secreted CAZy enzymes.

An increase in the expression of the endo-cellulase gene (AO090038000175) was then observed, peaking at ca. day 10. An endo-cellulase would likely be required in the early stages of cellulose degradation to cleave internal sites along the cellulose fibres and expose free reducing ends for exo-cellulases to attack, releasing lower molecular weight cello-oligomers (e.g. cellobiose) which in turn could be further depolymerised to glucose. This hypothesis was further supported by a similar degree of up-regulation of another gene with predicted cellulase activity (AO090005001553) from day 5 onwards as was seen with the endo-cellulase. From approximately day 5, a steady increase in the expression of a β-glucosidase gene (AO090113000148) was observed. This enzyme would act on the non-reducing ends of substrates created by endo-cellulase activities. Expression levels of the acetyl xylan esterase gene (*axeA*: AO090011000745) were also noted to increase steadily from day 5 onwards. This could indicate the increased expression of ‘scoping’ enzymes with potential to act on substrates present within the BSG (e.g. the side chain decorations of hemicelluloses) and thus liberate an additional metabolic carbon source. Perhaps at around this time, the *A. oryzae* was beginning to deplete all of the readily accessible carbon sources and a true starvation response was commencing. This may suggest that at this point, gluconeogenesis was occurring through the breakdown of less energetically favourable substrates such as the hordein-prolamin glycoproteins or their constituent glucogenic amino acids such as proline [32] or lignin. However, gluconeogenesis alone was not considered likely to explain the high ethanol yields seen with the best performing CBP permutations. Expression levels of the glucoamylase gene (*glaA*: AO090010000746) remained constant throughout the duration of the experiment suggesting that the *A. oryzae* was either not utilising the trace starch component of the BSG (as increased α-glycosidic bond hydrolysis would have indicated) or that it was occurring at a continually low, steady state.

### CBP Fermentation Optimisation

Since all experimental runs were conducted using static, semi-solid-state bioreactors (with high initial viscosity of the media), additional mixing could be employed in the future in an attempt to enhance fungal growth (and thus achieve greater enzyme secretion). This in turn might facilitate better enzyme-substrate interactions, potentially yielding greater fermentable sugar yields. The low solubility of oxygen (in water) relative to other dissolved solutes can limit aerobic fungal growth through limited oxygen mass transfer (especially in high dissolved solids bioreactors such as those employed here) and thus limit the secretion of various lignocellulolytic enzymes [33]. In addition, the mycelial growth of any filamentous fungal species in a solid state or high solids CBP reactor may increase the viscosity of the supernatant which may further limit oxygen mass transfer. Thus, the anaerobic environmental conditions generated by the sake CBP system (with either of the yeast strain variants tested) could have limited maximal ethanol yields, by restricting fungal growth (and thus activity of saccharification enzymes). However, the issue is likely to be considerably more complex and dynamic than can be explained by one single factor. Furthermore, both fungal hyphae and cellulases are sensitive to shear stresses, rendering agitation-based improvements in oxygen mass transfer challenging [33]. Micro-bubble-based dispersion systems could possibly be used, as opposed to conventional mixing protocols. Ideally, a dissolved oxygen concentration (DO_2_) of ≥20 % air saturation would be sufficient [34].

If one were to consider application of the sake CBP system for bioethanol production from BSG, it is of interest that other applications of *A. oryzae* might be developed. Since *A. oryzae* is able to decompose biodegradable plastic, such as poly-butylene succinate (PBS; [35]), it could perhaps be used in the recycling of biodegradable bottles. *A. oryzae* has both cutinase (*CutL1*) and hydrophobin genes (such as *rolA*) within its genome which have been shown to be responsible for the deconstructive mechanism. Cutinase facilitates the actual decomposition of the plastic, with hydrophobins acting as ‘scaffolding’ for the specific site recruitment of the cutinase onto the hydrophobic surfaces of biodegradable plastics [27, 35].

## Conclusions

The sake CBP system (*A. oryzae* and *S. cerevisiae* NCYC479) was by far the most effective of all permutations tested for ethanol production from BSG, with maximal ethanol yields of ca. 37 g/L produced within 10 days. On this basis, 94 kg of pure ethanol could be produced from 1 t of BSG using 36 hL water. Whilst volumetric productivity was moderate (3.7 g/L/day), the process requires no pre-treatment and no exogenous enzymes. The final waste residue contained >22 % crude protein. Utilising this co-product stream efficiently (e.g. as an animal feed) would further improve overall process economics.

## Electronic supplementary material

Supplementary Figure 1 Ethanol concentrations generated at various time points from consolidated bioprocessing of 50 g (dried and ground) BSG with 200 mL water at 15 °C and 30 °C using the sake based consortium of *A.oryzae* and *S.cerevisiae* NCYC479 with dried and ground BSG. Data are the mean ± SD of three replicate experiments. (DOC 199 kb)

Supplementary Figure 2 Composition of BSG used in the present study. Detected xylose concentrations liberated at various time points from a sake based system of consolidated bioprocessing of 50 g dried ground BSG (with 200 ml water) at both 15 °C and 30 °C using a consortium of *A.oryzae* and *S.cerevisiae* NCYC479 with and without the addition of Novozymes Cellic CTec2 (10 FPU/g biomass) on day 10. Data are the mean ± SD of three replicate experiments. (DOC 158 kb)

Supplementary Figure 3 Ethanol concentrations generated at various time points from consolidated bioprocessing of 50 g (dried and ground) BSG with 200 mL water and inoculated with only *Kluyveromyces spp*. and supplemented with Novozymes Cellic® CTec2 (10 FPU/g biomass) on day 10 (with no initial *S. cerevisiae* fermentation). Data are the mean ± SD of three replicate experiments. (DOC 247 kb)

Supplementary Figure 4 Ethanol concentrations generated at various time points from consolidated bioprocessing of 50 g (dried and ground) BSG with 200 mL water and at 25 °C using a consortium of *Aspergillus niger* and two separate strains of *Saccharomyces cerevisiae* (NCYC2592 or NCYC479)*.* Data are the mean ± SD of three replicate experiments. (DOC 76 kb)
