# Supplementary material for: Bioethanol Production from Brewers Spent Grains Using a Fungal Consolidated Bioprocessing (CBP) Approach
Source: Bioenergy Res. 2016 Aug 8;10(1):146–57. doi: 10.1007/s12155-016-9782-7 (PMC7114960; doi:10.1007/s12155-016-9782-7)
Supplement: Supplementary file 1 — Ethanol concentrations generated at various time points from consolidated bioprocessing of 50 g (dried and ground) BSG with 200 mL water at 15 °C and 30 °C using the sake based consortium of A.oryzae and S.cerevisiae NCYC479 with dried and ground BSG. Data are the mean ± SD of three replicate experiments. (DOC 199 kb) [file 12155_2016_9782_MOESM1_ESM.doc]

**Supplementary Figure 1:**


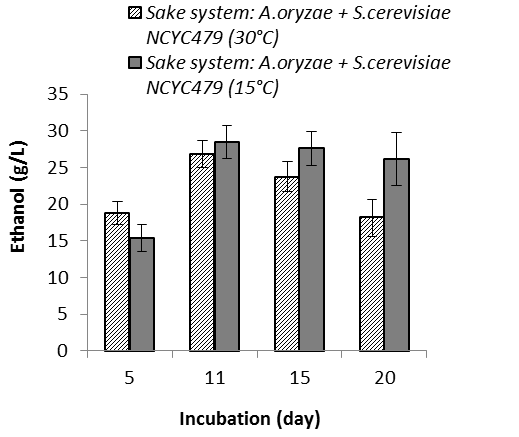


Ethanol concentrations generated at various time points from consolidated bioprocessing of 50 g (dried and ground) BSG with 200 ml water at 15°C and 30°C using the sake based consortium of *A.oryzae* and *S.cerevisiae* NCYC479 with dried and ground BSG. Data are the mean ± SD of three replicate experiments.
