# Supplementary material for: Bioethanol Production from Brewers Spent Grains Using a Fungal Consolidated Bioprocessing (CBP) Approach
Source: Bioenergy Res. 2016 Aug 8;10(1):146–57. doi: 10.1007/s12155-016-9782-7 (PMC7114960; doi:10.1007/s12155-016-9782-7)
Supplement: Supplementary file 2 — Composition of BSG used in the present study. Detected xylose concentrations liberated at various time points from a sake based system of consolidated bioprocessing of 50 g dried ground BSG (with 200 ml water) at both 15 °C and 30 °C using a consortium of A.oryzae and S.cerevisiae NCYC479 with and without the addition of Novozymes Cellic CTec2 (10 FPU/g biomass) on day 10. Data are the mean ± SD of three replicate experiments. (DOC 158 kb) [file 12155_2016_9782_MOESM2_ESM.doc]

**Supplementary Figure 2:**


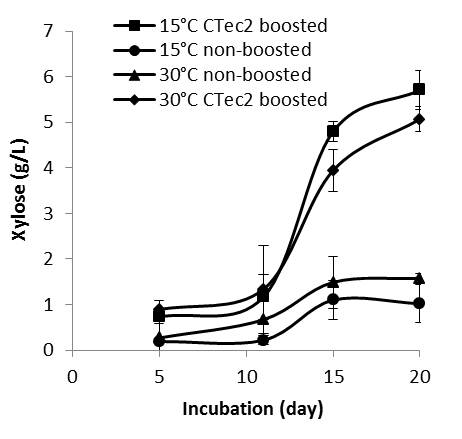


Detected xylose concentrations liberated at various time points from a sake based system of consolidated bioprocessing of 50 g dried ground BSG (with 200 ml water) at both 15°C and 30°C using a consortium of *A.oryzae* and *S.cerevisiae* NCYC479 with and without the addition of Novozymes Cellic CTec2 (10 FPU/g biomass) on day 10. Data are the mean ± SD of three replicate experiments.
