# Supplementary material for: Bioethanol Production from Brewers Spent Grains Using a Fungal Consolidated Bioprocessing (CBP) Approach
Source: Bioenergy Res. 2016 Aug 8;10(1):146–57. doi: 10.1007/s12155-016-9782-7 (PMC7114960; doi:10.1007/s12155-016-9782-7)
Supplement: Supplementary file 3 — Ethanol concentrations generated at various time points from consolidated bioprocessing of 50 g (dried and ground) BSG with 200 mL water and inoculated with only Kluyveromyces spp. and supplemented with Novozymes Cellic® CTec2 (10 FPU/g biomass) on day 10 (with no initial S. cerevisiae fermentation). Data are the mean ± SD of three replicate experiments. (DOC 247 kb) [file 12155_2016_9782_MOESM3_ESM.doc]

**Supplementary Figure 3:**


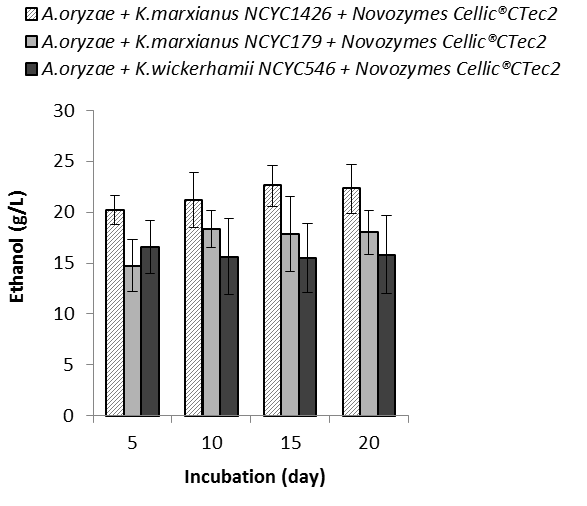


Ethanol concentrations generated at various time points from consolidated bioprocessing of 50 g (dried and ground) BSG with 200 ml water and inoculated with only *Kluyveromyces* *spp*. and supplemented with Novozymes Cellic® CTec2 (10 FPU/g biomass) on day 10 (with no initial *S. cerevisiae* fermentation). Data are the mean ± SD of three replicate experiments.
