# Supplementary material for: Bioethanol Production from Brewers Spent Grains Using a Fungal Consolidated Bioprocessing (CBP) Approach
Source: Bioenergy Res. 2016 Aug 8;10(1):146–57. doi: 10.1007/s12155-016-9782-7 (PMC7114960; doi:10.1007/s12155-016-9782-7)
Supplement: Supplementary file 4 — Ethanol concentrations generated at various time points from consolidated bioprocessing of 50 g (dried and ground) BSG with 200 mL water and at 25 °C using a consortium of Aspergillus niger and two separate strains of Saccharomyces cerevisiae (NCYC2592 or NCYC479). Data are the mean ± SD of three replicate experiments. (DOC 76 kb) [file 12155_2016_9782_MOESM4_ESM.doc]

**Supplementary Figure 4:**


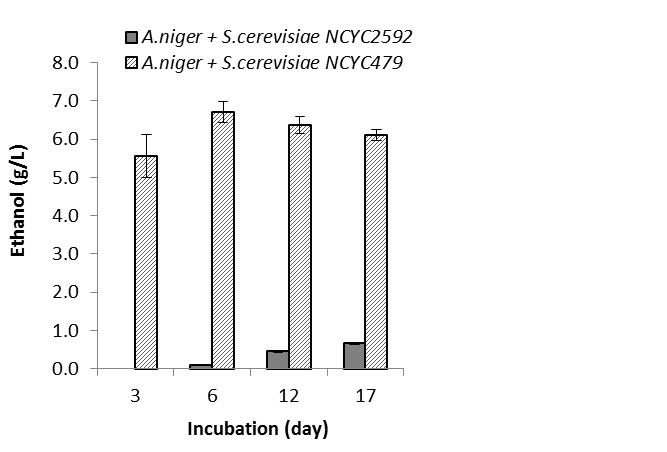


Ethanol concentrations generated at various time points from consolidated bioprocessing of 50 g (dried and ground) BSG with 200 ml water and at 25°C using a consortium of *Aspergillus niger* and two separate strains of *Saccharomyces cerevisiae* (NCYC2592 or NCYC479)*.* Data are the mean ± SD of three replicate experiments.
